# Supplementary material for: Expanding magnetic organelle biogenesis in the domain Bacteria
Source: Microbiome. 2020 Oct 30;8:152. doi: 10.1186/s40168-020-00931-9 (PMC7602337; doi:10.1186/s40168-020-00931-9)
Supplement: Supplementary file 2 — Additional file 1: Supplementary Table 1. Summary of sampled sites. [file 40168_2020_931_MOESM1_ESM.docx]

**Supplementary Table 1. Summary of sampled sites.**

| **Sample ID** | **Sample location** | **Environment** | **Latitude (°)** | **Longitude (°E)** | **Salinity (ppt)** | **pH** | **Reference** |
| --- | --- | --- | --- | --- | --- | --- | --- |
| ARSLQ | Innermogolia, China | Freshwater/Marginal | 47.19788 | 119.92877 | 0.3 | 6.8 | This study |
| Cal1 | Lake Catani, Australia | Freshwater/Marginal | -36.73426 | 146.81119 | <0.1 | / | Lin et al., 2018 |
| Cal2 | Lake Catani, Australia | Freshwater/Marginal | -36.73274 | 146.81226 | <0.1 | / | This study |
| CL | Corunna Lake, Australia | Freshwater/Marginal | -38.23694 | 144.42936 | 0.6 | / | This study |
| DC | Lake Dianchi, China | Freshwater/Marginal | 24.908133 | 102.74605 | 0.9 | 7.6 | Lin et al., 2018 |
| DC0425 | Lake Dianchi, China | Freshwater/Marginal | 24.908133 | 102.74605 | 0.9 | 7.6 | This study |
| DH2 | Lake East, China | Freshwater/Marginal | 30.555833 | 114.411389 | / | / | Zhang et al., 2020 |
| DJH13 | Peatland, Dajiu Lake, China | Acidic Peatland | 31.483672 | 110.000694 | <0.1 | 5.1 | This study |
| DJH14 | Peatland, Dajiu Lake, China | Acidic Peatland | 31.483503 | 110.000844 | <0.1 | 4.9 | This study |
| DJH15 | Peatland, Dajiu Lake, China | Acidic Peatland | 31.485181 | 110.001483 | <0.1 | 5.7 | This study |
| DJH2 | Peatland, Dajiu Lake, China | Acidic Peatland | 31.49194 | 109.99472 | <0.1 | 4.9 | This study |
| DJH5 | Peatland, Dajiu Lake, China | Acidic Peatland | 31.490422 | 109.995886 | 0.1 | 4.4 | This study |
| DJH6 | Peatland, Dajiu Lake, China | Acidic Peatland | 31.49055 | 109.99611 | 0.2 | 4.9 | This study |
| DJH8 | Peatland, Dajiu Lake, China | Acidic Peatland | 31.48944 | 109.99638 | <0.1 | 4.3 | This study |
| ER1 | Erskine River, Australia | Brackish/Saline/Marine | -38.5338 | 143.97832 | 3.2 | / | Lin et al., 2018 |
| ER2 | Erskine River, Australia | Brackish/Saline/Marine | -38.5359 | 143.97478 | 1.9 | / | Lin et al., 2018 |
| GR | Gellibrand River, Australia | Brackish/Saline/Marine | -38.69715 | 143.15477 | 1.3 | / | This study |
| HA1 | Pond, Hongan, China | Freshwater/Marginal | 31.295 | 114.916944 | 0.4 | 7.3 | This study |
| HA3d | Pond, Hongan, China | Freshwater/Marginal | 31.173819 | 114.547642 | 0.2 | 5.2 | Lin et al., 2018 |
| HA4 | Pond, Hongan, China | Freshwater/Marginal | 31.292222 | 114.921667 | 0.3 | 7.3 | This study |
| HA5a | Pond, Hongan, China | Freshwater/Marginal | 31.290556 | 114.913333 | 0.4 | 7.5 | This study |
| HAa3 | Rice field, Hongan, China | Freshwater/Marginal | 31.174289 | 114.543941 | <0.1 | 5.2 | Lin et al., 2018 |
| HCH | Xi’an city moat, China | Freshwater/Marginal | 34.25287 | 108.92187 | 0.2 | 7.5 | Lin et al., 2018 |
| HGR | Honggaori Lake, Inner Mogolia, China | Freshwater/Marginal | 41.573047 | 108.313566 | 0.4 | 8.0 | This study |
| HLH | Hulun Lake, Inner Mogolia, China | Freshwater/Marginal | 49.06944 | 117.75694 | 0.7 | 8.6 | This study |
| JC1 | Pond, Inner Mogolia, China | Freshwater/Marginal | 41.687541 | 108.393862 | 0.3 | 8.2 | This study |
| JC2 | Pond, Inner Mogolia, China | Freshwater/Marginal | 41.686682 | 108.39418 | 0.3 | 8.2 | This study |
| JSW | Jinshawan, Yingkou, China | Brackish/Saline/Marine | 40.223972 | 122.083844 | / | / | This study |
| KLK | Keluke Lake, Qinghai, China | Brackish/Saline/Marine | 37.283722 | 96.859816 | 4.2 | 7.9 | This study |
| MBP | Mount Beauty Pondage, Australia | Freshwater/Marginal | -36.73874 | 147.16443 | <0.1 | / | Lin et al., 2018 |
| MY | Lake Miyun, China | Freshwater/Marginal | 40.48874 | 117.00714 | 0.2 | 7.5 | Lin et al., 2018 |
| N2-2 | Naritu, Inner Mogolia, China | Freshwater/Marginal | 41.549487 | 109.05952 | 0.3 | 7.5 | This study |
| N3 | Naritu, Inner Mogolia, China | Freshwater/Marginal | 41.549487 | 109.05952 | 0.8 | 7.8 | This study |
| NGH | Nuogan Lake, Inner Mogolia, China | Freshwater/Marginal | 47.9275 | 119.54638 | 0.3 | 7.4 | This study |
| PC | Painkalac Creek, Australia | Brackish/Saline/Marine | -38.46575 | 144.09288 | 21.7 | / | Lin et al., 2018 |
| PCR | Punkally Creek, Australia | Brackish/Saline/Marine | -36.23413 | 150.06798 | 33.9 | / | Lin et al., 2018 |
| QXH1 | Qixian Lake, Inner Mogolia, China | Freshwater/Marginal | 47.74777 | 119.33694 | 0.7 | 8.4 | This study |
| QXH2 | Qixian Lake, Inner Mogolia, China | Freshwater/Marginal | 47.69541 | 119.30422 | 0.3 | 8.5 | This study |
| S315 | Lake, Qinghai, China | Brackish/Saline/Marine | 38.229771 | 90.697681 | 4.3 | 8.5 | This study |
| SSYD | Shuishangyadan, Qinghai, China | Brackish/Saline/Marine | 37.624414 | 93.733812 | 15.3 | 7.6 | This study |
| TS | Tuosu Lake, Qinghai, China | Brackish/Saline/Marine | 37.197953 | 96.868083 | 3.8 | 7.8 | This study |
| W3 | Wulantaolegai, Inner Mogolia, China | Freshwater/Marginal | 41.564702 | 109.081861 | 0.4 | 8.1 | This study |
| W5 | Wulantaolegai, Inner Mogolia, China | Freshwater/Marginal | 41.564702 | 109.081861 | 0.4 | 8.1 | This study |
| wag | Waganaga Inlet, Australia | Brackish/Saline/Marine | -36.21831 | 150.12168 | 36.5 | / | This study |
| wal | Wallaga Lake, Australia | Brackish/Saline/Marine | -36.36013 | 150.07384 | 37.0 | / | This study |
| WMH | Lake Weiming, China | Freshwater/Marginal | 39.993142 | 116.30256 | 0.3 | 7.5 | Lin et al., 2018 |
| WRX1 | Weierxun river, Inner Mogolia, China | Freshwater/Marginal | 48.23222 | 117.62305 | 0.2 | 8.0 | This study |
| WRX2 | Weierxun river, Inner Mogolia, China | Freshwater/Marginal | 48.23111 | 117.62277 | 0.3 | 7.9 | This study |
| WRX3 | Weierxun river, Inner Mogolia, China | Freshwater/Marginal | 48.455 | 117.63305 | 0.2 | 7.9 | This study |
| XX | Aiken, creek, Qinghai, China | Freshwater/Marginal | 38.170074 | 90.57854 | 0.8 | 7.7 | This study |
| YD0423 | Yuandadu Park, China | Freshwater/Marginal | 39.974732 | 116.368688 | 0.5 | 7.2 | This study |
| YD0425 | Yuandadu Park, China | Freshwater/Marginal | 39.974732 | 116.368688 | 0.5 | 7.2 | Lin et al., 2018 |
| YQH56 | Yanqi Lake, Beijing, China | Freshwater/Marginal | 40.39833 | 116.67777 | / | / | This study |

Lin W, Zhang W, Zhao X, et al. Genomic expansion of magnetotactic bacteria reveals an early common origin of magnetotaxis with lineage-specific evolution. ISME J, 2018, 12:1508–1519.

﻿Zhang W, Ji R, Liu J, et al. Two metagenome-assembled genome sequences of magnetotactic bacteria in the order *Magnetococcales*. Microbiol Resour Announc 2020, 9:7-9.
