# Supplementary material for: Expanding magnetic organelle biogenesis in the domain Bacteria
Source: Microbiome. 2020 Oct 30;8:152. doi: 10.1186/s40168-020-00931-9 (PMC7602337; doi:10.1186/s40168-020-00931-9)
Supplement: Supplementary file 4 — Additional file 3: Supplementary Table 3. Previously published MTB genomes included in this study. [file 40168_2020_931_MOESM3_ESM.docx]

**Supplementary Table 3. Previously published MTB genomes included in this study.**

| **Organism name** | **GenBank accession number** |
| --- | --- |
| Alphaproteobacteria bacterium WMHbin7 | PDZW00000000 |
| Candidatus Lambdaproteobacteria bacterium PCRbin3 | PDZZ00000000 |
| Candidatus Magnetaquicoccus inordinatus strain UR-1 | RXIU00000000 |
| Candidatus Magnetobacterium bavaricum isolate TM-1 | LACI00000000 |
| Candidatus Magnetobacterium casensis strain MYR-1 | JMFO00000000 |
| Candidatus Magnetococcus massalia MO-1 | JN177318 |
| Candidatus Magnetoglobus multicellularis str. Araruama | ATBP00000000 |
| Candidatus Magnetominusculus xianensis strain HCH-1 | LNQR00000000 |
| Candidatus Magnetomorum sp. HK-1 | JPDT00000000 |
| Candidatus Magnetoovum chiemensis strain CS-04 | JZJI00000000 |
| Candidatus Omnitrophica bacterium isolate Cal1bin1 | PEAR00000000 |
| Candidatus Omnitrophica bacterium isolate MBPbin6 | PEAF00000000 |
| Candidatus Omnitrophus magneticus isolate SKK-01 | JYNY00000000 |
| Candidatus Terasakiella magnetica strain PR-1 | FLYE00000000 |
| Deltaproteobacteria bacterium isolate ER2bin7 | PEAL00000000 |
| Deltaproteobacteria bacterium YD0425bin50 | PDZT00000000 |
| Deltaproteobacteria bacterium YD0425bin51 | PDZS00000000 |
| Desulfamplus magnetovallimortis strain BW-1 | FWEV00000000 |
| Desulfovibrio magneticus RS-1 | NC_012796 |
| Ectothiorhodospiraceae bacterium BW-2 | CP032507 |
| Latescibacteria bacterium SCGC AAA252-B13 | ASWY00000000 |
| Magnetococcales bacterium DC0425bin3 | PEAP00000000 |
| Magnetococcales bacterium DCbin2 | PEAO00000000 |
| Magnetococcales bacterium DCbin4 | PEAN00000000 |
| Magnetococcales bacterium HA3dbin3 | PEAJ00000000 |
| Magnetococcales bacterium HCHbin5 | PEAG00000000 |
| Magnetococcales bacterium isolate ER1bin7 | PEAM00000000 |
| Magnetococcales bacterium isolate HA3dbin1 | PEAK00000000 |
| Magnetococcales bacterium isolate HAa3bin1 | PEAI00000000 |
| Magnetococcales bacterium isolate WMHbin1 | PDZY00000000 |
| Magnetococcales bacterium isolate YD0425bin7 | PDZU00000000 |
| Magnetococcales bacterium WMHbin3 | PDZX00000000 |
| Magnetococcales bacterium WMHbinv6 | PDZV00000000 |
| Magnetococcus marinus MC-1 | NC_008576 |
| Magnetofaba australis IT-1 | LVJN00000000 |
| Magnetospira sp. QH-2 | FO538765 |
| Magnetospirillum caucaseum strain SO-1 | AONQ00000000 |
| Magnetospirillum gryphiswaldense strain MSR-1 | NC_023065 |
| Magnetospirillum kuznetsovii strain LBB-42 | PGTO01000000 |
| Magnetospirillum magneticum strain AMB-1 | NC_007626 |
| Magnetospirillum magnetotacticum MS-1 | JXSL00000000 |
| Magnetospirillum marisnigri strain SP-1 | LWQT00000000 |
| Magnetospirillum moscoviense strain BB-1 | LWQU00000000 |
| Magnetospirillum sp. ME-1 | CP015848 |
| Magnetospirillum sp. XM-1 | LN997848 |
| Magnetovibrio blakemorei strain MV-1 | MCGG00000000 |
| Nitrospira bacterium SG8_35_4 | LJTM00000000 |
| Nitrospirae bacterium isolate DC0425bin1 | PEAQ00000000 |
| Nitrospirae bacterium isolate HCHbin1 | PEAH00000000 |
| Nitrospirae bacterium isolate MYbin2 | PEAE00000000 |
| Nitrospirae bacterium MYbin3 | PEAD00000000 |
| Nitrospirae bacterium MYbin6 | PEAC00000000 |
| Nitrospirae bacterium MYbinv3 | PEAB00000000 |
| Omnitrophica WOR_2 bacterium GWA2_45_18 | MHFX01000000 |
| Omnitrophica WOR_2 bacterium GWC2_45_7 | MHGD01000000 |
| Planctomycetes bacterium SM23_25 | LJTY00000000 |
| Terasakiella sp. SH-1 | CP038255 |
| Uncultured Desulfobacteraceae bacterium isolate CR-1 | CAACVI000000000 |
| Zetaproteobacteria bacterium isolate PCbin4 | PEAA00000000 |
