# Supplementary figures and images for: Expanding magnetic organelle biogenesis in the domain Bacteria
Source: Microbiome. 2020 Oct 30;8:152. doi: 10.1186/s40168-020-00931-9 (PMC7602337; doi:10.1186/s40168-020-00931-9)

MTB genomes reconstructed in this study

Previously published MTB genomes

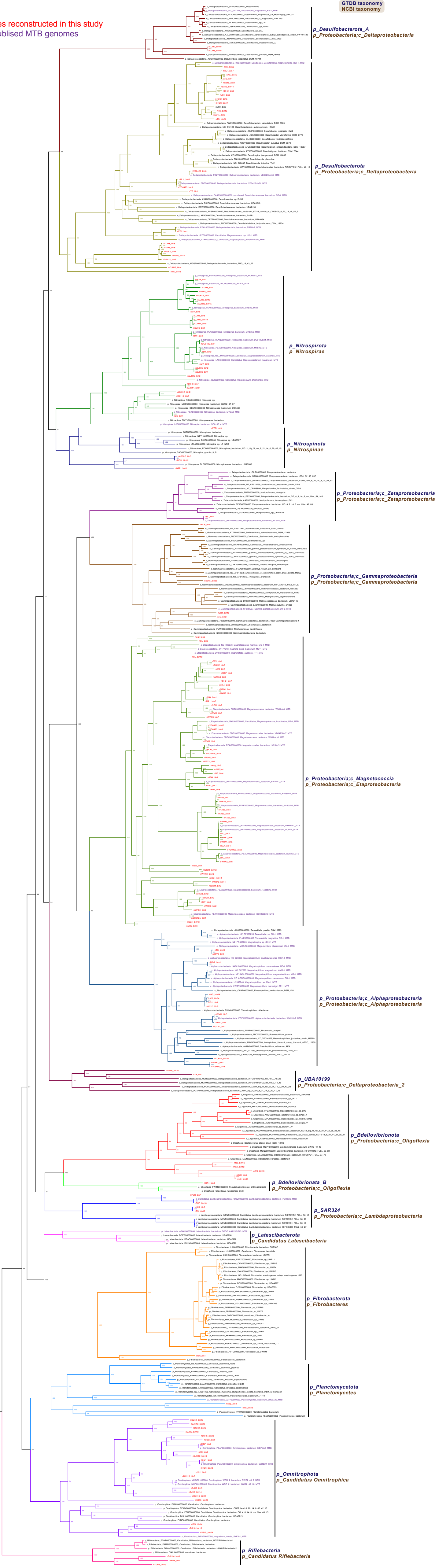

Supplement: Supplementary file 5 — Additional file 4: Supplementary Figure 1. Maximum likelihood phylogenomic tree of MTB genomes and their close non-MTB relatives. [file 40168_2020_931_MOESM4_ESM.pdf]

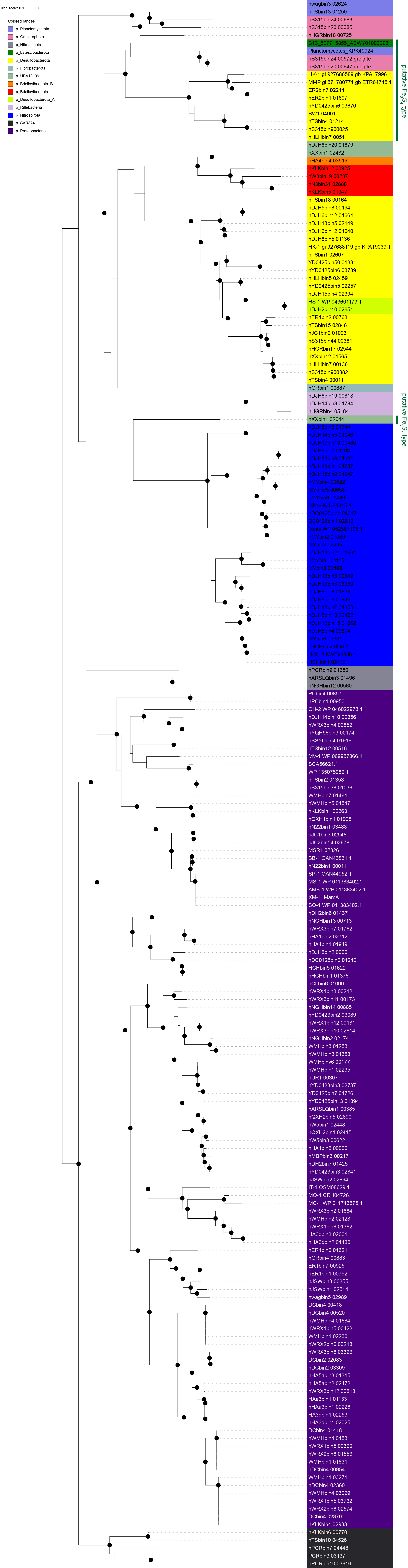

Supplement: Supplementary file 6 — Additional file 5: Supplementary Figure 2. Maximum-likelihood tree of magnetosome protein MamA. [file 40168_2020_931_MOESM5_ESM.jpg]

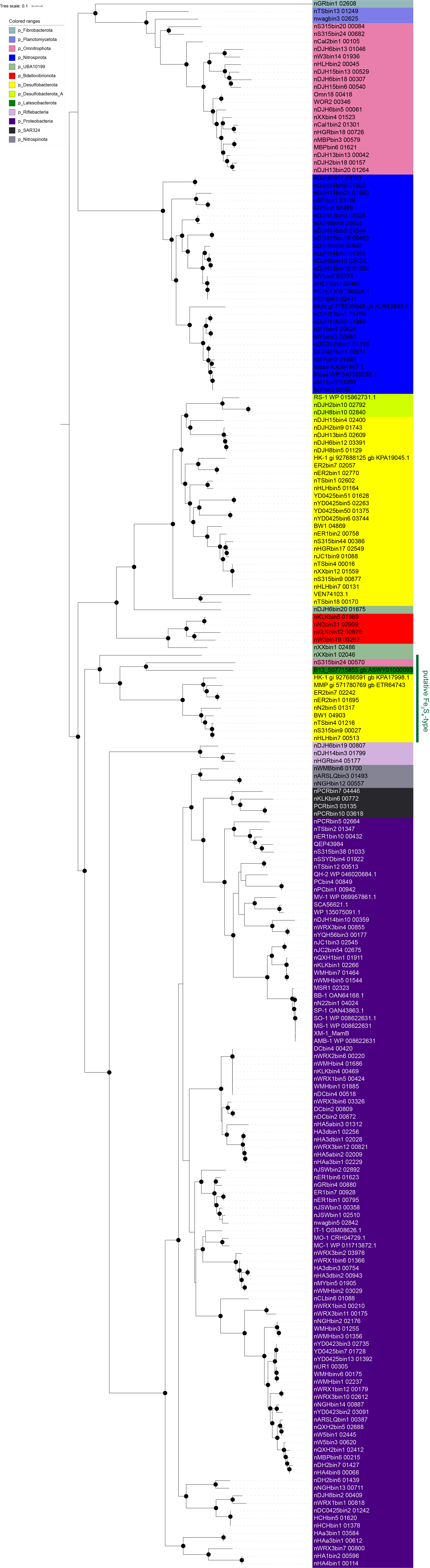

Supplement: Supplementary file 7 — Additional file 6: Supplementary Figure 3. Maximum-likelihood tree of magnetosome protein MamB. [file 40168_2020_931_MOESM6_ESM.jpg]

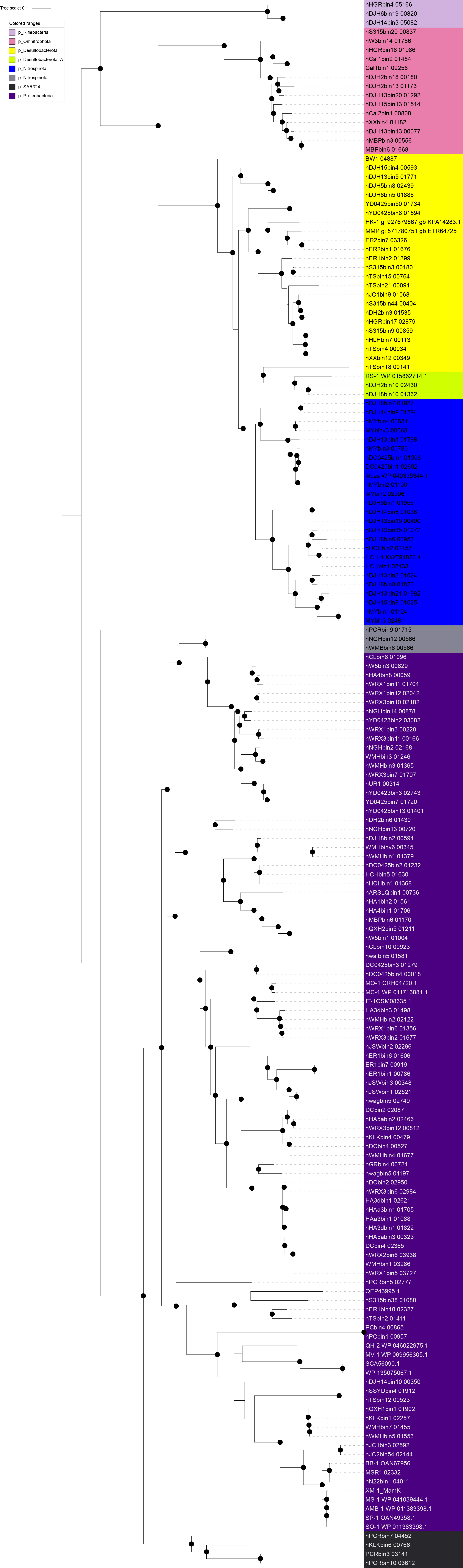

Supplement: Supplementary file 8 — Additional file 7: Supplementary Figure 4. Maximum-likelihood tree of magnetosome protein MamK. [file 40168_2020_931_MOESM7_ESM.jpg]

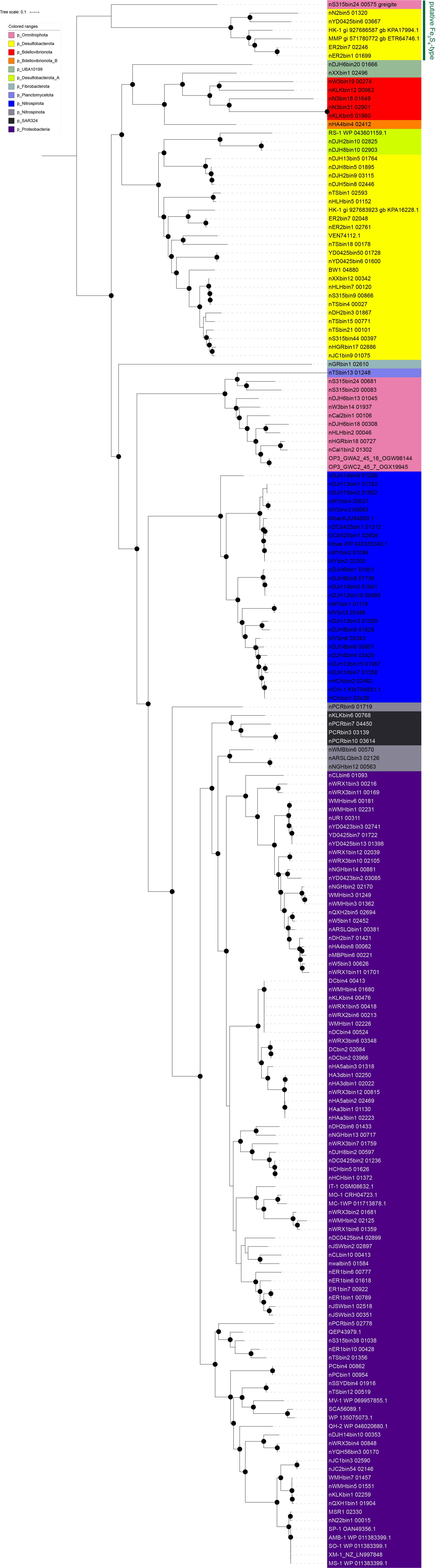

Supplement: Supplementary file 9 — Additional file 8: Supplementary Figure 5. Maximum-likelihood tree of magnetosome protein MamM. [file 40168_2020_931_MOESM8_ESM.jpg]

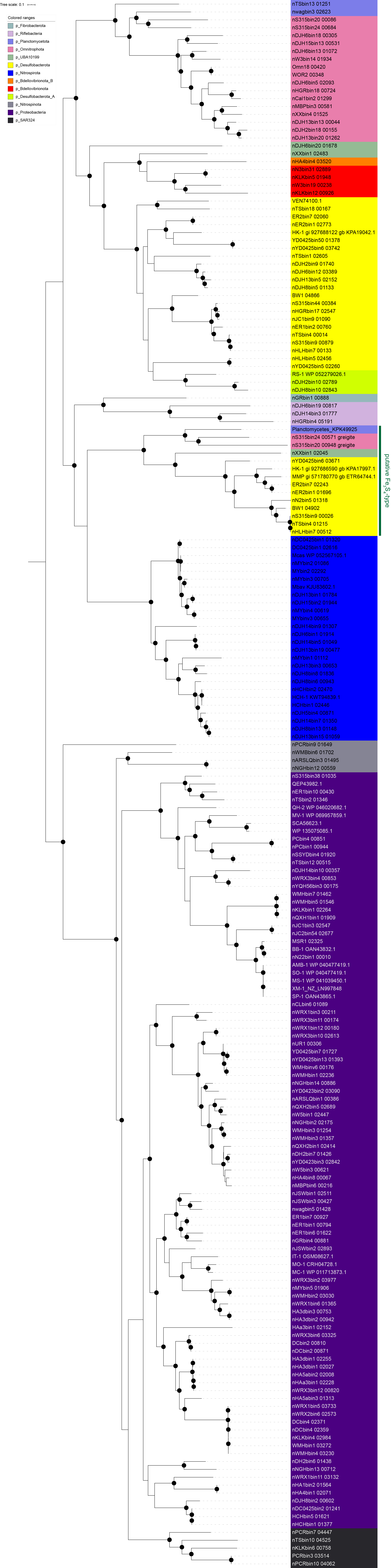

Supplement: Supplementary file 10 — Additional file 9: Supplementary Figure 6. Maximum-likelihood tree of magnetosome protein MamQ. [file 40168_2020_931_MOESM9_ESM.jpg]

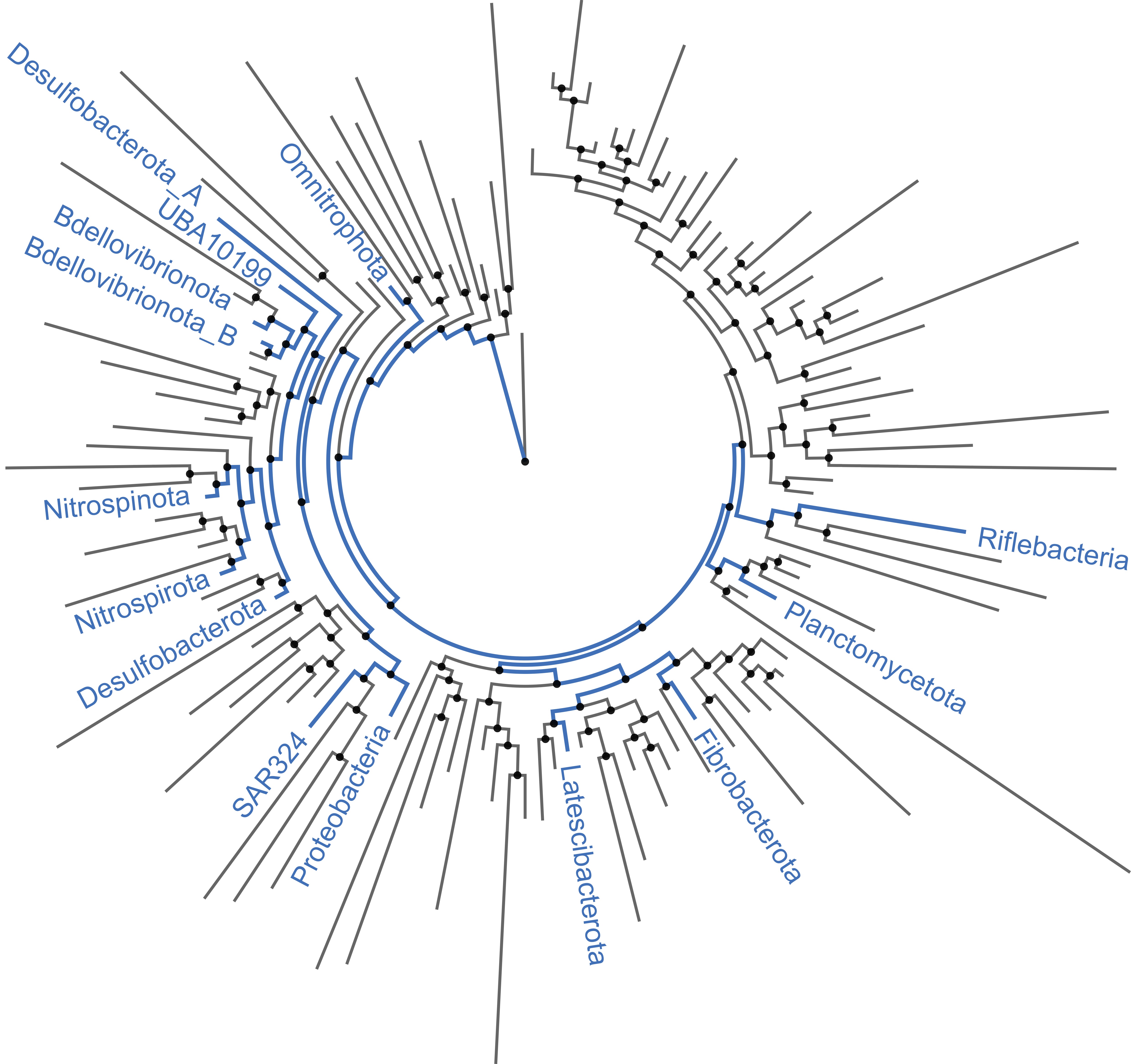

Supplement: Supplementary file 11 — Additional file 10: Supplementary Figure 7. Phylogenetic distribution of MTB-containing phyla across the Bacterial tree of life. The phylum level Bacterial tree of life with MTB-containing phyla highlighted in blue. The Bacterial tree was made using the AnnoTree server. [file 40168_2020_931_MOESM10_ESM.jpg]
